# Comparing Facets of Emotion Dysregulation as Daily Cannabis Use Predictors: A Dominance Analysis Study

**DOI:** 10.21203/rs.3.rs-10371817/v1

**Published:** 2026-07-27

**Authors:** MeKayla Smith, Samuel J. West, Larry Keen

**Affiliations:** Virginia State University

**Keywords:** Cannabis, Emotion, Dominance Analysis, Regulation, Impulsivity

## Abstract

Cannabis remains the most widely used illicit substance in the United States, specifically among young adults. Previous research identifies emotion dysregulation as a predictor of cannabis use, yet there remains some uncertainty in the literature regarding which facets of emotion dysregulation are the most important in relation to cannabis use. Indeed, the studies that have compared the facet-level associations of dysregulation with cannabis use have provided broadly conflicting results. These conflicts in the literature may be due, in part, to researchers relying on simple comparisons of correlation or regression coefficients; a problematic approach given the correlations among the facets of dysregulation. This study applied a regimen of model selection and dominance analyses in order to provide some clarity regarding which facets of emotion dysregulation are the most important predictors of cannabis use.

**Method:**

A sample of 353 undergraduate students completed an online survey study that included the Difficulties in Emotion Regulation Scale (DERS) and the Daily Sessions, Frequency, Age of Onset, and Quantity of Cannabis Use Inventory.

**Results:**

Statistical analyses, including negative binomial regression and dominance analysis, indicated that the DERS impulsivity facet as the most dominant predictor of daily cannabis use, followed by the strategies and non-acceptance facets, respectively.

**Conclusion:**

These findings underscore the importance of targeting specific components of emotion regulation difficulties in interventions aimed at reducing cannabis use among young adults. We offer suggestions for the integration of our findings in the development of targeted interventions.

## Introduction

Cannabis is the most commonly used illicit substance in the United States (Baldwin et al., 2024) and has been increasing globally among both adolescents and adults (Pessar et al., 2025). As of June 2025, recreational cannabis use has been legalized in 24 states and the District of Columbia in the United States (Murphy, 2021). Recent estimates indicate that over 52 million Americans have used cannabis at least once in their lifetime (Centers for Disease Control and Prevention, 2025). As cannabis use prevalence continues to rise in the general population, there are concerns regarding the effect cannabis use has on an individual's health (Hasler et al., 2025). These health concerns include psychological and behavioral functions, potentially due to the cannabis-related alterations within the central nervous system (Lu, 2021; Mackenzie, 2023). These cannabis-related alterations are associated with the endocannabinoid system, which include central autonomic brain regions that are associated with emotion regulation (Meyer, 2018; Sharkey, 2016).

Previous research posits individuals who use cannabis are more likely to experience internalizing symptoms such as anxiety and other forms of distress (Choi, 2023). Further, researchers report a direct link between emotion dysregulation and problematic cannabis use (Cavalli & Cservenka, 2021; Buckner et al., 2024). Individuals who use cannabis are more likely to experience difficulties in regulating their emotions compared to individuals who do not (Stellern, 2023). Cannabis users that experience emotion dysregulation are more likely to employ maladaptive strategies when dealing with negative stressors or emotional stimuli, which may lead to increases in cannabis use and cannabis use severity-related problems (Buckner et al., 2017). Moreover, chronic cannabis users may experience more internalizing symptoms in comparison to those who do not use cannabis (Keen et al., 2023). As such, the literature suggests that emotion dysregulation is both a risk factor for and a consequence of problematic cannabis use.

Previous research evinces a positive link between emotion dysregulation problematic substance use generally (e.g., Garke et al., 2021) and problematic cannabis use specifically (e.g., Cavalli & Cservenka, 2021). The association between cannabis use and emotion dysregulation may be due to individuals using cannabis as an external means of emotion regulation under stressful situations. Indeed, individuals who struggle with cessation of cannabis use cite difficulties with emotion regulation as a common reason for relapse (Buckner et al., 2017). The Difficulties with Emotion Regulation Scale (DERS; Gratz et al., 2004) is the single most used measure of emotion dysregulation in the literature (Stellern et al., 2023). The DERS measures emotion dysregulation as a total score comprising six lower-order facets: impulsivity in the face of negative emotions (impulsivity), lack of emotion regulation strategies (strategies), negative metacognitions about emotional states (non-acceptance), disruptions in goal-oriented behavior due to emotions (goals), unawareness of emotional states (awareness), and the lack of understanding of emotional experiences (clarity). These lower-order facets of difficulties in emotion regulation are also meaningful independent predictors of cannabis use (Buckner et al., 2017). Identifying the facets of emotion regulation most strongly related to relevant outcomes such as substance use may play a crucial role in developing treatment plans.

Despite the greater resolution afforded by breaking constructs down to the facet level, this literature has provided conflicting accounts of which facet of emotion dysregulation is most strongly linked with substance use and cannabis use specifically. Meta-analytic evidence suggests that all facets of emotion dysregulation are significantly higher in substance abusing populations, but that the strategies and impulsivity facets demonstrate the largest differences between groups (Stellern et al., 2023). More recent work indicates that all facets of the DERS are greater in participants with SUD (excepting the non-acceptance facet), with the clarity facet showing the largest difference between groups (Mansueto et al., 2024). Research comparing facets of the DERS as predictors of cannabis use has indicated that non-acceptance is the only significant predictor of coping-motivated cannabis use specifically in when the other facets of emotion dysregulation are accounted for (Wisener & Khoury, 2020). Further work indicates that the non-acceptance facet predicts coping as a key motive for smoking cannabis, but that none of the DERS facets predict the frequency use directly (Bonn-Miller et al., 2008). In contrast, some studies report that the non-acceptance facet of the DERS is the only facet that does not significantly predict cannabis use over the past month (Weidberg et al., 2023). Another study comparing the DERS facets between alcohol users who also smoke cannabis against those who do not reported that the non-acceptance, strategies, and impulsivity facets were all higher in cannabis users, with the strategies facet showing the largest difference (Moskal et al., 2023).

Such conflicting results in the scientific literature that center around comparing the strength of the facets of emotion dysregulation may be due to analytical shortcomings.

Studies that have compared emotion regulation facets as predictors of substance use outcomes commonly compare zero-order correlations or standardized beta coefficients and the associated 95% confidence intervals resulting from different steps of hierarchical multiple regression models or meta-analytic effect estimates (e.g., Stellern et al., 2023). Although common in the literature, these simple analytic approaches are inadequate for establishing the relative importance among correlated predictors. For example, the order in which variables are entered into a hierarchical regression is deterministic given that conclusions drawn from them may differ based on such order. Some studies that draw contrasts between the facets of the DERS in predicting substance use fail to include the variables from previous model steps (e.g., Bonn-Miller et al., 2008) or include the facets in separate path models (e.g., Weidberg et al., 2023) further obscuring the ability to draw valid comparisons. Further, variables that are highly correlated at the zero-order level like the facets from the DERS raise concerns of multicollinearity in multiple regression models which may produce unreliable results (e.g., Short et al., 2016; Azen & Budescu, 2006). Dominance analysis (Azen & Budescu, 2003) provides an analytic framework as an answer to these limitations of traditional regression approaches.

Dominance analysis allows researchers to establish relative importance of predictors while controlling multicollinearity by estimating the average levels of contribution for each predictor variable across every possible level of model complexity (i.e., the number of predictors in the model) and every possible combination of predictors (Azen & Budescu, 2003; Azen & Budescu, 2006). The dominance analysis framework tests variables for three levels of dominance that vary in their strictness: complete, conditional, and general dominance in the prediction of a given outcome (e.g., violent injuries; McLaurin et al., 2025). Complete dominance reflects the strictest level of dominance and indicates that there were no model cases in which any other variable was a stronger predictor of the DV. Conditional dominance tests the relative importance of predictors at each model of level complexity and allows the researcher to explore how complexity may impact the average contributions of each predictor. Finally, general dominance is established by a simple comparison of the overall average coefficient of determination values for each predictor across all models tested.

### Current Study

The frequency of cannabis use is positively associated with difficulty in emotion regulation. Although some research has examined this association at the facet level, these studies have provided conflicting findings. One possible explanation for these conflicts in the literature could be the inherent shortcomings of applying less appropriate statistical approaches to relative importance. For example, the facets of emotion dysregulation are highly colinear, making the traditional comparison of standardized beta coefficients in a multiple regression model challenging to interpret due to the violation of this assumption (multicollinearity) made by most regression models. The current study presents a regimen of exploratory dominance analyses to identify the facet(s) of emotion dysregulation that are most strongly linked with cannabis use in a sample of young adults. We developed no hypotheses a priori for the current study as the conflicting findings in the literature made any such predictions difficult to justify.

## Methods

### Participants

The current study included 353 undergraduate young adults that were recruited from an Historically Black University on the east coast. Participants were conveniently sampled from undergraduate courses through flyers and professor referrals for a parent study. Participants were recruited on campus through flyers, the use of the SONA System, and professor referrals. Inclusion criteria for the current study included individuals between the age of 18–25 and must be an undergraduate student. Participants in this sample were 20.75 years old on average, *SD* = 4.79). Women constituted 76.60% of our sample. Among participants in this sample, 91% identified as African American/Black, 2% White, 0.80% Hispanic or Latino, 0.40% Indigenous American, and the remaining 5.50% identified as mixed race or ‘other’. This study has received approval from the university’s Institutional Review Board.

### Measures

Daily Cannabis Use. We relied on a single-item assessment of the daily frequency of cannabis use from the Daily Sessions, Frequency, Age of Onset, and Quantity of Cannabis Use Inventory (DFAQ-CU; Cuttler et al., 2017). Specifically, we asked respondents to report “How many times a day, on a typical weekday, do you use cannabis?” as an open-ended response.

Difficulty with Emotion Regulation. The Difficulties in Emotion Regulation Scale (DERS) was used to assess an individual's emotion regulation (Gratz et al., 2004). The DERS includes 36 questions to assess difficulties with emotion regulation across six facets (non-acceptance, goals, awareness, strategies, impulse, and clarity). The non-acceptance subscale measured the extent to which individuals have negative meta-emotions about current mood-states (e.g., “When I'm upset, I become angry with myself for feeling that way”). The goals subscale assessed difficulties maintaining focus and engaging in goal-directed behavior when emotionally distressed (e.g., “When I'm upset, I have difficulty getting work done"). The awareness subscale evaluated how attentive the respondent is to their emotional states (e.g., “I pay attention to how I feel”). The strategies subscale measures limited access to practical coping methods or emotion regulation strategies (e.g., “When I'm upset, I believe there is nothing I can do to make myself feel better”). The impulse subscale gauges difficulties controlling impulsive behaviors during negative emotions (e.g., “When I’m upset, I become out of control.”). Finally, the clarity subscale assessed difficulty recognizing and understanding one’s emotions (e.g., “I have difficulty making sense of my feelings”). Each of the six facets were measured using six items each. Participants rated each item on a 5-point Likert scale in terms of how frequently each of the statements applied to them on a scale of 1 (almost never) to 5 (almost always), with higher scores indicating greater difficulties in emotion regulation.

### Procedure

The first page of the online assessment provided informed consent information to the participant, requiring the participant to type in their first and last names to indicate consent. After providing informed consent, each participant completed a battery of measures via a Qualtrics online survey. The survey obtained self-reported demographic information, substance use behaviors and a variety of psychosocial measures. These measures included Difficulties in Emotion Regulation Scale and Daily Sessions, Frequency, Age of Onset, and Quantity of Cannabis Use Inventory. After completion of the online survey, participants’ names were collected and recorded so that they may receive extra credit in their respective courses as compensation for their participation.

### Statistical Analyses

All statistical analyses were completed using R version 4.4.0 (R Core Team, 2024). We used the MASS package and base R functions to estimate Gaussian, Poisson, and negative binomial regression models for model selection (Venables & Ripley, 2002). We compared our models using the AIC, BIC, and rmse base R functions. We also extracted sigma estimates as a measure of fit from our models and examined their accuracy in accounting for observed zeros using the performance package (Ludecke et al., 2021). Our final dominance analysis was conducted using the suite of functions in the dominance analysis package (Navarrete & Soares, 2024).

## Results

### Statistical Power

We used G*power version 3.1.9.7 (Faul et al., 2009) for Windows to conduct a sensitivity power analysis for the most complex models we planned to estimate (i.e., all six facets of the DERS predicting daily cannabis sessions). Results of this analysis revealed that our sample of 353 allowed us to estimate multiple regression models with six predictors that explain approximately 4% or more in the dependent variable (i.e., R^2^ = .04 or greater) at 80% power.

### Descriptives

Descriptive statistics for all study variables are presented in Table 1along with internal consistency values (via McDonald’s ω) and the number of univariate outliers for each. All of the facets of the DERS demonstrated sufficient internal reliability and were roughly normal per the observed skew and kurtosis values. One participant reported levels of daily cannabis use that marked them as an extreme outlier in our sample (i.e., 8.45 standard deviations above the mean) and were thus excluded from all analyses. Our variable indexing the number of times participants consumed cannabis on a typical weekday demonstrated severe deviations from normality and overdispersion (i.e., the standard deviation was more than double the mean). This feature of our DV was expected as it is common for variables measured by counting the number of times a behavior occurs to exhibit zero inflation. This was indeed the case for our DV as 268 of our 359 participants reported zeros.

The zero-order correlations between all variables in the current study are reported in Table 2. Given the heavily skewed and ordinal nature of our DV and the mild skew in some of the DERS facets we report Spearman’s rank-order correlations. The clarity, goals, and impulsivity facets from the DERS were each positively associated with cannabis use and one another. The remaining DERS facets demonstrated significant positive correlations between facets ranging from weak to strong.

### Regression Models

Prior to conducting our focal dominance analysis, we estimated three regression models in order to find the model that best fit our data given the non-normal distribution of our outcome variable. Specifically, we estimated Gaussian, Poisson, and negative binomial models. We then compared the model fit indices from the resultant models to select the distribution that best fit our data (Table 3).

### Model RMSE Sigma AIC BIC

Our Gaussian regression model demonstrated considerably poorer fit relative to the Poisson and negative binomial models. Our RMSE values were generally similar across models, but the AIC and BIC values indicated that the negative binomial model provided a better fit than the Gaussian or Poisson models. We then compared the Poisson and negative binomial models in terms of their accuracy in accounting for the zeros in our dataset. The negative binomial model correctly predicted 99% of the observed zeros where the Poisson model only predicted 84% of the observed zeros correctly. As such, we selected the negative binomial model given its superior fit on multiple indices and accuracy in estimation for our zero-inflated DV. The initial results of the full multiple regression can be found in Table 4. This model accounted for 10% of the variance in daily cannabis uses per Nagelkerke’s R^2^. Impulsivity related to negative emotions was the only significant predictor of the number of daily cannabis sessions in our initial model.

Consistent with the zero-order correlations found between DERS facets, the Strategies facet of the DERS demonstrated moderate levels of variance inflation and thus ostensible multicollinearity.

### Dominance Analysis

Complete Dominance. Our negative binomial model was used as the basis of our planned dominance analyses. We examined the complete, conditional, and general dominance of the facets of the DERS in predicting cannabis use frequency. The results from our complete dominance analysis are displayed in Table 5. We were unable to identify a single completely dominant facet of the DERS as the impulsivity and strategies facets each exhibited complete dominance over the goals and awareness facets, but neither dominated one another. We thus proceeded to review the conditional dominance results.

Conditional Dominance. Conditional dominance allows researchers to examine the average contributions of each predictor to explaining the DV across varying levels of model complexity (i.e., the number of IVs in each model). Our conditional dominance analysis results are depicted in Table 6. One clear change was noted between our complete and conditional dominance models: the impulsivity facet conditionally dominated all other facets of the DERS. No other facets exhibited dominance patterns that differed from those found at the complete dominance stage.

**Figure 1:**
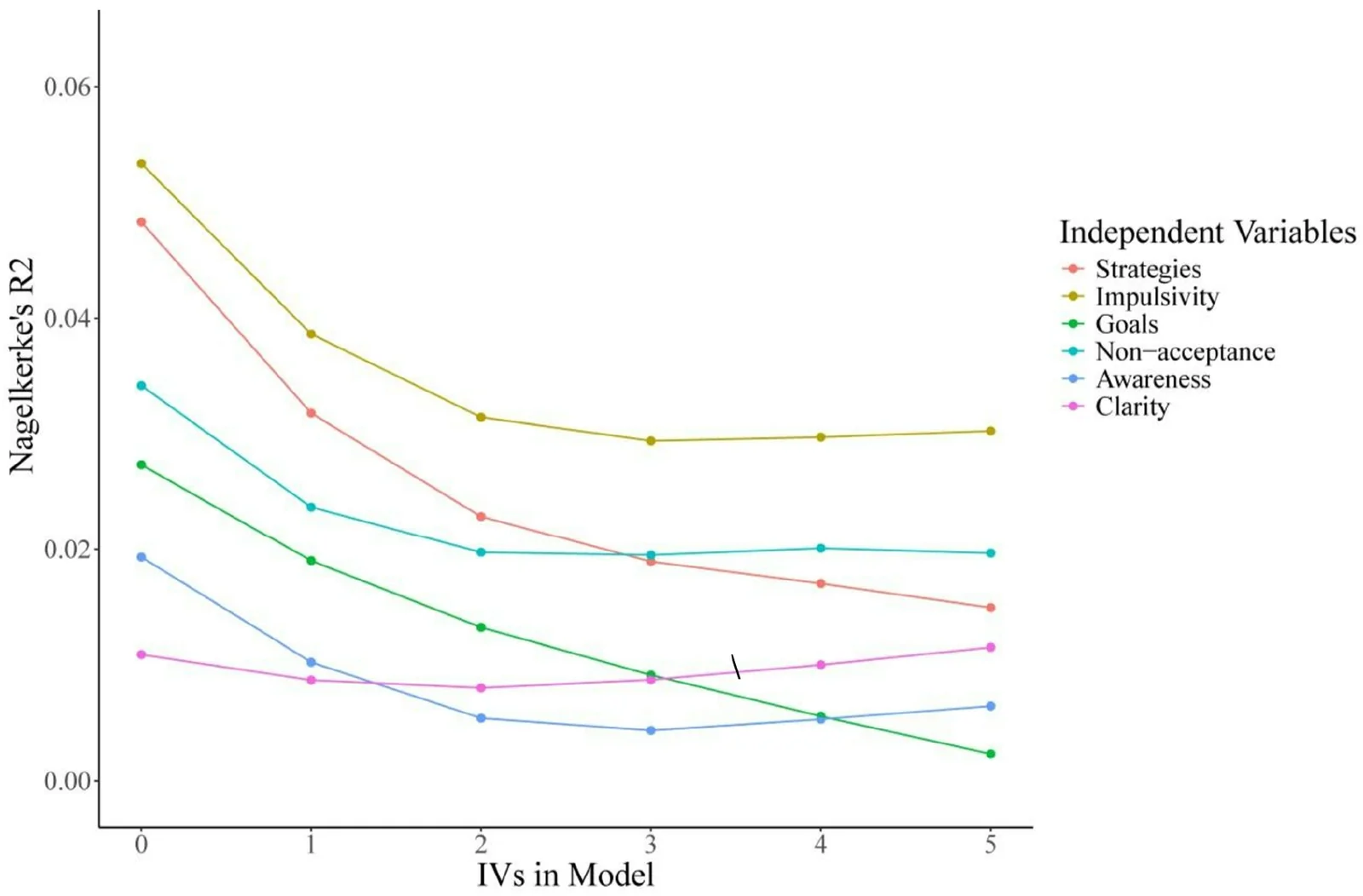
Conditional Dominance Plot Showing the Average Contribution of Each IV Across Every Level of Model Complexity

Overall, we found that at the lowest level of complexity (i.e., only one predictor) impulsivity and strategies were generally the strongest correlates of cannabis use (Fig. 1). However, the proportion of variance explained by these predictors decreased sharply with the inclusion of more IVs. This trend stabilized at the second level of complexity for the impulsivity facet, but the strategies facet continued to lose explained variance as model complexity increased. until the fourth level of model complexity after which they stabilized (i.e., did not lose more explained variance). The other DERS facets each explained some variation at the lowest level of complexity but reached levels near to zero once the complexity increased. Similarly, the clarity facet of the DERS explained next to no variance in cannabis use across all levels of model complexity.

General Dominance. Although our conditional dominance results provided greater clarity to our results, some ambiguity remained. As such we proceeded to review the general dominance results. The general dominance analysis results are presented in Table 7. The impulsivity facet of the DERS exhibited general dominance over all other DERS facets in predicting cannabis use frequency followed by the strategies facet which dominated all others excepting impulsivity. The non-acceptance facet exhibited a dominance pattern consistent with the previous analysis steps as it dominated the goals, awareness, and clarity facets. Of the remaining three facets, goals dominated awareness and clarity, clarity dominated awareness, and awareness dominated no other facets.

**Figure 2:**
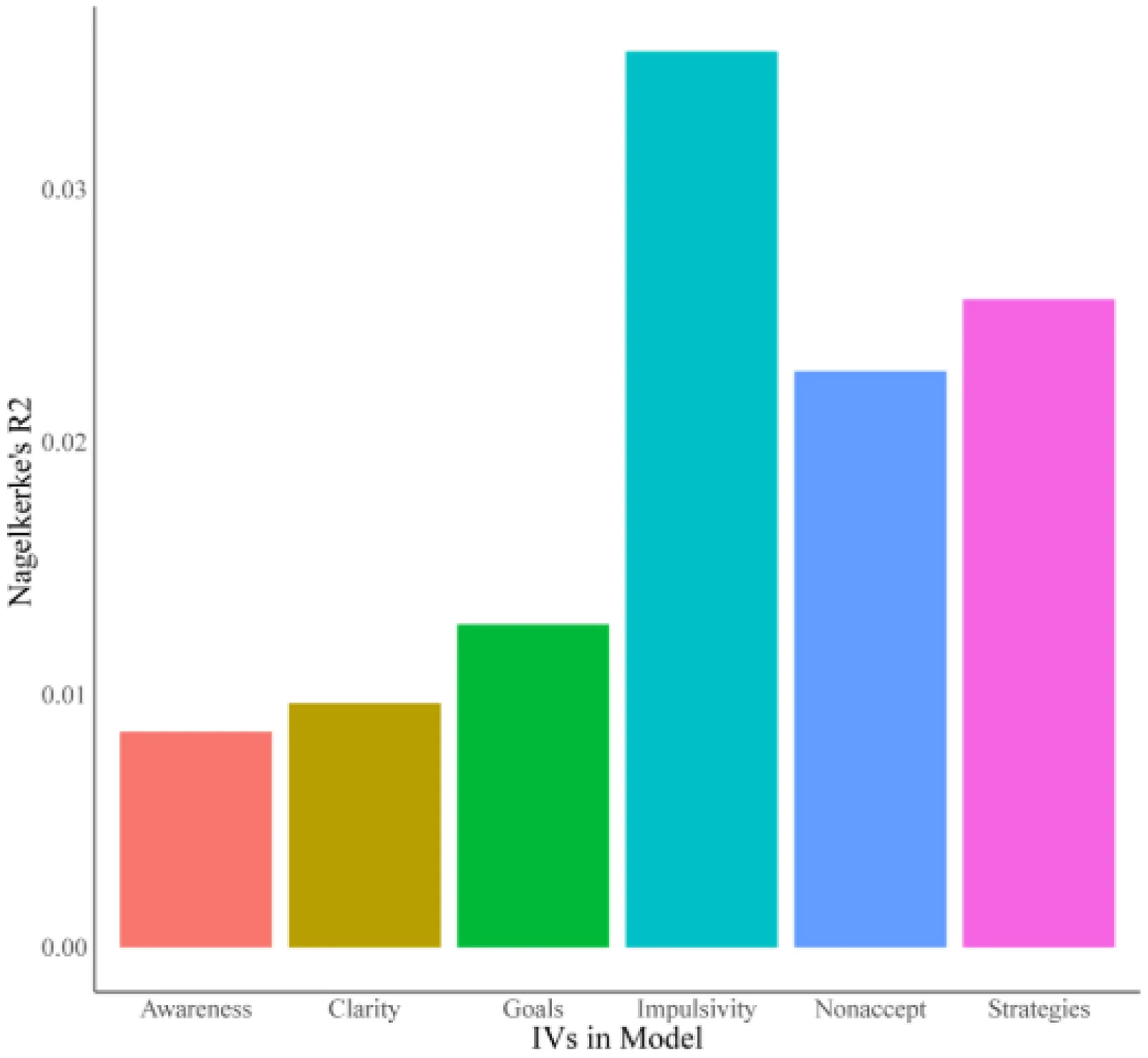
General Dominance Plot Depicting the Average Contribution of all Predictors Regardless of Model Complexity

Largely consistent with the complete and conditional dominance analyses, the general dominance results revealed impulsivity, strategies, and non-acceptance as the most important predictors of cannabis use in terms of the variance explained (Fig. 2). Among these three, non-acceptance was the weakest correlate, followed by the strategies facet, whereas the impulsivity facet clearly explained more variance in cannabis use frequency than any other facet from the DERS.

## Discussion

The current study explored the relationship between daily cannabis use and the facets of difficulties with emotion regulation through a dominance analysis approach. Our zero-order correlations revealed that three facets of emotion dysregulation (impulsivity, goals, and clarity) were positively associated with cannabis use frequency. However, our multiple regression containing all facets of the DERS as predictors indicated that only the impulsivity was a significant predictor of cannabis use. Our dominance analyses revealed that the impulsivity, strategies, and non-acceptance facets dominated the other facets when predicting cannabis use frequency, whereas impulsivity emerged as the single most dominant predictor when considering the results across the complete, conditional, and general levels of our dominance analysis.

### Difficulty with Emotion Regulation and Cannabis Use

Results of the current study indicate three facets of the DERS are particularly relevant in predicting cannabis use. Arranged in terms of dominance, the impulsivity, strategies, and non-acceptance facets each accounted for unique variance and dominated the other facets. The impulsivity facet of the DERS was the most important predictor of cannabis use frequency as it explained the most variance in cannabis use frequency compared to the other DERS facets at the general level and dominated all other facets at the conditional level. The strategies facet emerged as the second most dominant predictor at each level of dominance and was tied with impulsivity and non-acceptance for complete dominance. The non-acceptance facet was the third-most dominant predictor, as it dominated all facets except strategies and impulsivity at the conditional and general dominance levels.

### Addressing Conflicting Results

Despite the clear results provided by our regimen of analyses our results also partially conflict with much of the literature that has drawn similar comparisons. Our finding that the impulsivity facet was the single most dominant predictor of cannabis use frequency is consistent with previous work indicating impulsivity as the strongest predictor of substance use (Estévez et al., 2017) and risky behavior more generally (Dingle et al., 2018). This finding is also consistent with recent research indicating that higher levels of emotion dysregulation and impulsivity are risk factors for problematic cannabis use specifically (Vieira, 2024). In contrast, other evidence indicates the strategies facet as the most important facet for substance abuse (Stellern et al., 2023). Such findings conflict with our result for the impulsivity facet, but not our findings that the strategy facet was the second most important predictor of cannabis use. In similar conflict with our findings, other work demonstrates a larger difference in the strategies facet than the impulsivity facet when compared between participants with living with a substance use disorder and controls (Mansueto et al., 2024). However, our findings are consistent with meta-analytic evidence indicating that those with living with a substance use disorder have significantly greater levels of all DERS facets compared to healthy controls, but the differences are greatest for the strategies, impulse, and non-acceptance facets (Stellern et al., 2023).

### Implications

Our results hold several critical implications for the study of cannabis and substance use more broadly in the context of emotion regulation. Taken together our results point towards a specific subset of emotion regulation difficulties as being relevant to cannabis use. The impulsivity, strategies, and non-acceptance facets of the DERS were the most dominant predictors of cannabis use and showed strong, positive associations with one another. It may thus be the case that all three of these facets play different roles in motivating cannabis users to rely on cannabis for regulation of their negative emotional states. For example, a frequent cannabis user may become angry due to some interpersonal provocation. A common impulse in such scenarios is to act aggressively as a means of mood repair (e.g., Chester & DeWall, 2017).

However, aggression carries considerably more risk than consuming cannabis, especially in environments where cannabis use is legalized. In this context cannabis may be used strategically to protect dysregulated users against more harmful impulses, rendering them less able to regulate such emotions without cannabis in the future. Here the non-acceptance facet of emotion dysregulation may maintain or elevate the initial pangs of negative affect which may lead to an even greater desire to consume cannabis. This interpretation is supported by evidence indicating that cannabis reduces state anger above and beyond alcohol (Trull et al., 2016). Other work with clinical populations (e.g., bi-polar disorder patients) indicates that frequent cannabis users have a greater baseline level of anger relative to controls, but that anger levels are indistinguishable from controls after consuming cannabis (Gruber et al., 2012). Our findings also highlight the importance of developing tailored interventions that target the specific features of emotion dysregulation among those struggling with CUD, so as to reduce the risk of cannabis use.

Clinical efficacy studies show that emotion regulation skills can be broadly improved by standardized clinical therapeutic approaches (e.g., dialectical and cognitive behavioral therapies) that focus on providing the client with effective strategies for dealing with difficult feelings (Paucsik, 2024; Saccaro, 2024). However, these therapies commonly focus on some of the aspects of dysregulation that aren’t especially relevant to treating substance use outcomes. For example, awareness and clarity are both a major focus in cognitive behavioral therapy approaches that utilize mindfulness training techniques. In contrast, there is little intervention literature that specifically targets the metacognitive aspect of emotional non-acceptance, which our results suggest may be an important target for treatment in CUD patients. Future work should aim to develop more specific therapeutic frameworks that bolster the impulse control, self-acceptance, and regulation strategy features of emotion regulation in individuals to reduce the risk of cannabis use in order to lay the groundwork for a greater capacity for intrinsic emotion regulation.

Our findings also demonstrate the importance of utilizing appropriate analyses for examining the relative strength of predictors. Our zero-order associations suggested that the impulsivity, goals, and clarity facets were the only ones associated with cannabis use. However, our regimen of dominance analyses made clear that the goals and clarity facets were less important predictors than those that were not significant at the zero-order level (i.e., the strategies and non-acceptance facets). This pattern of results suggests that the significant zero-order associations found between the clarity and goals facets with cannabis use were spurious and due entirely to the overlapping variances they shared with the impulsivity facet.

### Limitations and Future Directions

The current study carries several important limitations. First, our study was exploratory and used a cross-sectional design, which limited our ability to determine causality or directionality between cannabis use and emotion regulation. Future work should test these associations and probe their temporal dynamics through longitudinal and ecological momentary assessment designs. Second, our measure of emotion regulation only assessed aspects of regulating emotional states with negative valence. Although it is true that only negative emotions are necessarily distressing, a sizable literature on contra-hedonic motivation indicates that people also engage in the downregulation of positive emotions in various instances (e.g., Riediger et al., 2009). Impulsivity, the most important predictor of cannabis use in the current study, is especially relevant in this context given the body of research indicating positive urgency (i.e., reacting impulsively to positive emotions) as a strong predictor of antisocial behavior more broadly (e.g., West et al., 2023). Similarly, the DERS did not allow us to compare cannabis’ effects on emotional states that carry different levels of arousal (e.g., boredom versus anger).

Future work should examine how cannabis’ links with emotion regulation affects the entire breadth of human emotion rather than focusing solely on emotions with negative valence and high arousal. Finally, the current study comprised a sample of college undergraduates who were subclinical in their difficulties with emotion regulation. These effects should be replicated with samples of participants who present clinically relevant levels of these behavioral and emotional difficulties.

## Conclusion

The current study sought to resolve ambiguity in the literature regarding which facet of emotion dysregulation shares the strongest relationship with cannabis use. Results of our dominance analysis regimen indicated that the impulsivity facet of the DERS was the most important predictor of cannabis use frequency. However, the strategies and non-acceptance facets also emerged as important, but weaker, predictors of cannabis use. Our findings suggest that the relationship between emotion dysregulation and cannabis use is driven by the impulsivity, strategies, and non-acceptance facets. Our findings also underscore the importance of using dominance analysis when examining the relative importance of multicollinear predictors in correlational studies. Cannabis researchers interested in understanding which of a set of correlated predictors is the most strongly linked to their outcome of interest should include dominance analysis as a central feature of their analytic toolkit.

## Figures and Tables

**Table 1:** Descriptive and Reliability Statistics of All Study Variables

Awareness	M (SD)	Range	Skew	Kurtosis	ω	Outliers
	14.57 (5.62)	6–30	0.33	−0.59	0.84	0
Clarity	11.32 (4.13)	5–22	0.27	−0.69	0.76	0
Cannabis Use	0.49 (1.01)	0–5	2.41	5.93	-	10
Goals	13.40 (5.05)	5–25	0.33	−0.54	0.87	0
Impulse	12.11 (4.85)	6–30	1.08	1.13	0.84	6
Non-acceptance	11.78 (5.64)	6–30	1.00	0.38	0.90	2
Strategies	17.07 (6.42)	8–39	0.80	0.40	0.85	3
Zero-Order Correlations						

**Table 2:** Zero-order Spearman’s Rank-Order Correlations Among All Study Variables

1Awareness	1	2	3	4	5	6
	-					
2Cannabis Use	−.02	-				
3Clarity	.56***	.13*	-			
4Goals	.12*	.11*	.41***	-		
5Impulsivity	.25***	.16**	.51***	.53***	-	
6Non-acceptance	.25***	.06	.54***	.50***	.60***	-
7Strategies	.26***	.08	.60***	.63***	.71***	.75***

Note.

* p < .05

** p < .01

*** p < .001

**Table 3:** Comparison of All Regression Models

NB	0.99	1.00	635.68	666.61
Gaussian	0.98	0.99	1003.10	1034.04
Poisson	0.98	1.00	716.28	743.35

Note. AIC = Akaike information criterion, BIC = Bayesian information criterion, RMSE = root mean square error.

**Table 4:** Results from the Negative Binomial Regression Model

Predictor	IRR	CI	p	VIF
Awareness	0.96	0.92–1.01	.127	1.40
Clarity	1.08	1.00–1.17	.060	2.17
Goals	1.03	0.97–1.08	.376	1.79
Impulsivity	1.10	1.04–1.17	.003	2.12
Non-acceptance	0.96	0.92–1.01	.216	2.50
Strategies	0.95	0.90–1.03	.260	3.81

Note. IRR = Incidence Rate Ratio, VIF = Variance Inflation Factor.

**Table 5:** Matrix of Complete Dominance Results

1 Strategies	1	2	3	4	5	6
	-	0.5	1	0.5	1	0.5
2 Impulsivity	0.5	-	1	0.5	1	1
3 Goals	0	0	-	0.5	0.5	0.5
4 Non-acceptance	0.5	0.5	0.5	-	1	0.5
5 Awareness	0	0	0.5	0	-	0.5
6 Clarity	0.5	0	0.5	0.5	0.5	-

Note. Dominance of row variables over columns is indicated by 1, dominance of column variables over rows is indicated by 0, variable pairs where dominance could not be established are indicated with 0.5.

**Table 6:** Matrix of Conditional Dominance Results

1 Strategies	1	2	3	4	5	6
	-	0	1	0.5	1	1
2 Impulsivity	1	-	1	1	1	1
3 Goals	0	0	-	0	0.5	0.5
4 Non-acceptance	0.5	0	1	-	1	1
5 Awareness	0	0	0.5	0	-	1
6 Clarity	0	0	0.5	0	0.5	-

Note. Dominance of row variables over columns is indicated by 1, dominance of column variables over rows is indicated by 0, variable pairs where dominance could not be established are indicated with 0.5.

**Table 7:** Matrix of General Dominance Results

1 Strategies	1	2	3	4	5	6
	-	0	1	1	1	1
2 Impulsivity	1	-	1	1	1	1
3 Goals	0	0	-	0	1	1
4 Non-acceptance	0	0	1	-	1	1
5 Awareness	0	0	0	0	-	0
6 Clarity	0	0	0	0	1	-

Note. Dominance of row variables over columns is indicated by 1, dominance of column variables over rows is indicated by 0.

## Data Availability

The datasets generated and/or analyzed during the current study are not publicly available because they contain information that could compromise participant privacy and/or are subject to institutional or ethical restrictions. De-identified data may be made available from the corresponding author upon reasonable request and with approval from the relevant ethics committee.
